# A High-Molecular-Weight Fraction of Planarian Mucus Triggers UPR-Linked Cell Death Pathway in Human Bronchioalveolar Carcinoma Cell Line NCI-H358

**DOI:** 10.3390/ijms27104324

**Published:** 2026-05-12

**Authors:** Gaetana Gambino, Gemma Marcelli, Paola Iacopetti, Laura Benvenuti, Chiara Bertini, Lucia Giambastiani, Luisa Pozzo, Alessandra Salvetti, Leonardo Rossi

**Affiliations:** 1 Department of Clinical and Experimental Medicine, University of Pisa, Via Volta 4, 56126 Pisa, Italy; gemma.marcelli@med.unipi.it (G.M.); paola.iacopetti@unipi.it (P.I.); chiara.bertini@unitn.it (C.B.); leonardo.rossi@unipi.it (L.R.); 2 Department of Translational Research and New Technologies in Medicine and Surgery, University of Pisa, Via Savi 10, 56126 Pisa, Italy; laura.benvenuti@phd.unipi.it; 3 Department of Physics, University of Trento, Via Sommarive 14, 38123 Trento, Italy; 4 Institute of Agricultural Biology and Biotechnology (IBBA), National Research Council (CNR), Via Moruzzi 1, 56124 Pisa, Italy; lucia.giambastiani@ibba.cnr.it (L.G.); luisa.pozzo@cnr.it (L.P.)

**Keywords:** natural bioactive compounds, anticancer agent, *Dugesia japonica*, mucus, unfolded protein response

## Abstract

Natural products remain a major source of anticancer agents, yet freshwater organisms are largely unexplored. Building on our previous evidence that planarian mucus exerts cytostatic and cytotoxic effects on cancer cells, we investigated the involvement of endoplasmic reticulum stress and unfolded protein response (UPR) pathways. Mucus-induced cytotoxicity is ROS-dependent and associated with depletion of intracellular reduced glutathione (GSH), not through inhibition of the System Xc^−^ transporter but potentially associated with upregulation of CHAC1, a glutathione-degrading enzyme. Mucus fractionation based on molecular weight identified the high-molecular-weight crude fraction as the one containing the bioactive entity, reproducing the effects of whole mucus. Treatment with this fraction early activates the PERK–ATF4 branch of the UPR, which could be responsible for driving CHAC1 induction. Moreover, ATF4 enhances DDIT3 expression, and activates a compensatory NRF2-dependent antioxidant response. At a later stage mucus also activates the IRE1α–XBP1 axis, with no ATF6 involvement, indicating selective UPR engagement in response to oxidative and lipid stress. Overall, our data are consistent with a potential PERK–ATF4–CHAC1–GSH self-sustaining axis promoting oxidative stress that culminates in cell death, supporting the potential of planarian mucus as a source of pleiotropic bioactive compounds, although the molecular identity of the active component(s) remains still unresolved.

## 1. Introduction

Bioprospecting remains a cornerstone of anticancer agent discovery—natural products and their derivatives constitute the majority of approved chemotherapeutic small molecules [1].

Classic examples include camptothecin (from *Camptotheca acuminata*), paclitaxel (from *Taxus brevifolia*), and the vinca alkaloids (from *Catharanthus roseus*) [2,3].

Marine bioprospecting further expanded the drug-lead repertoire, drawing from sponges, tunicates, and associated microorganisms [4].

Although terrestrial and especially marine programs are extensive, bioprospecting of freshwater animal taxa remains comparatively underdeveloped. Indeed, annual marine surveys report >1400 new structures, whereas freshwater systems have yielded only sporadic discoveries, including antimicrobial peptides from amphibians (*Xenopus laevis* and *Phyllomedusa* spp.) [5,6], piscidins from freshwater fishes (*Morone* and *Oreochromis* spp.) [7,8], bioactive metabolites from the microbiome of *Spongilla lacustris* [9], and the anticoagulant peptide hirudin from the leech *Hirudo medicinalis* [10].

Among the few freshwater-derived findings, the identification of a planarian extract exhibiting cytostatic and cytotoxic effects against human cancer cell lines [11] provided the conceptual basis for our investigation into the molecular effects of planarian mucus as a potential source of anti-neoplastic compounds.

Building on this evidence, we have recently demonstrated that mucus collected from *Dugesia japonica* (Platyhelminthes, class Turbellaria, order Tricladida) inhibits cancer cell growth by activating cytostatic and reactive oxygen species (ROS)-dependent cytotoxic mechanisms, including lipid droplet accumulation, dissipation of mitochondrial transmembrane potential, and mitochondrial membrane reorganization [12]. Ultrastructural analyses further revealed that mucus treatment can trigger multiple modes of cell death, notably ferroptosis and apoptosis [12].

In the present work, we found that a high-molecular-weight fraction of planarian mucus activates a self-sustaining stress circuit involving PERK–ATF4–CHAC1 signaling, unfolded protein response (UPR) pathways, and glutathione (GSH) depletion, leading to sustained oxidative stress.

## 2. Results

### 2.1. Active Cytotoxic Fraction of Mucus Has a Molecular Weight Higher than 100 kDa

**Figure 1:**
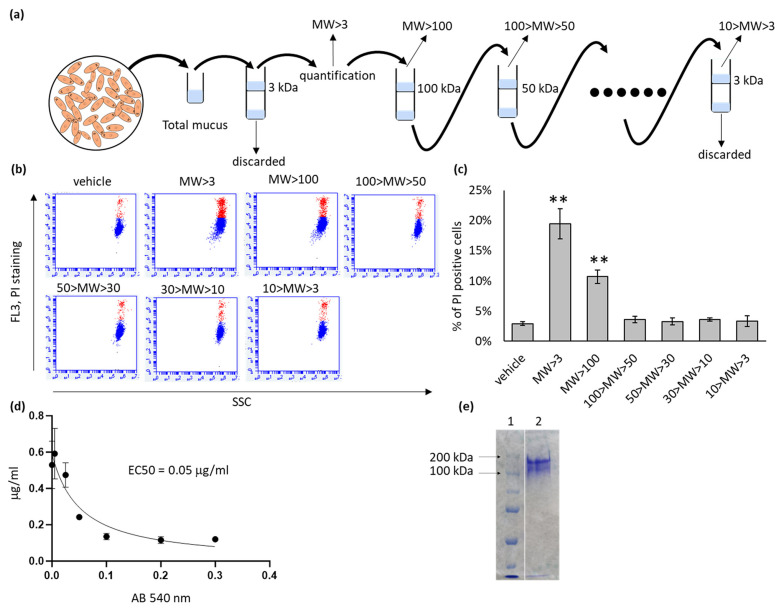
Fractionation and cytotoxic activity of planarian mucus. (**a**) Schematic representation of the mucus fractionation procedure. (**b**) Representative flow cytometry plots from the PI exclusion assay. Events considered as PI-positive are stained in red. SSC, side scatter channel. (**c**) Quantification of cell death (% of PI-positive cells) detected in a representative experiment for different mucus fractions, 48 h after treatment. Each bar represents the mean ± SD of five independent samples. ** = *p* < 0.01 calculated using one-way ANOVA. MW > 3 corresponds to total mucus prepared according to Gambino et al. [12]. (**d**) EC_50_ value of the MW > 100 mucus fraction determined by crystal violet assay (n = 3). (**e**) Coomassie Brilliant Blue staining of the MW > 100 mucus fraction separated by SDS–PAGE. Lane 1: MW marker; lane 2: MW > 100 mucus fraction.

To identify the fraction of planarian mucus responsible for cytotoxic activity, we fractionated mucus preparations obtained as described by Gambino and colleagues [12] according to the molecular weight (MW) of their components, using a series of protein concentrators as outlined in Figure 1a.

Before fractionation, the total mucus volume was measured and quantified based on its polysaccharide content, following the method reported by Gambino and co-workers [12], to determine the appropriate microliter amount to be added per milliliter of treatment medium.

After each fractionation step, the mucus volume was restored to the original value, and an equivalent amount (*v*/*v*) of each fraction was used to treat cells. A total of 48 h after treatment, the percentage of dead cells was evaluated using the propidium iodide (PI) exclusion assay.

As shown in Figure 1b,c, cytotoxic activity was mainly retained in the highest molecular weight fraction (MW > 100 kDa, from this point onward called HMW-MF). To standardize subsequent treatments, polysaccharides in HMW-MF were quantified, and its EC_50_ was calculated through the crystal violet assay (Figure 1d).

Coomassie Brilliant Blue staining (Figure 1e) revealed two predominant protein bands within this fraction, with estimated molecular weights between 150 and 200 kDa.

### 2.2. HMW-MF Induces GSH Depletion Through an Xc^−^-Independent Mechanism

**Figure 2:**
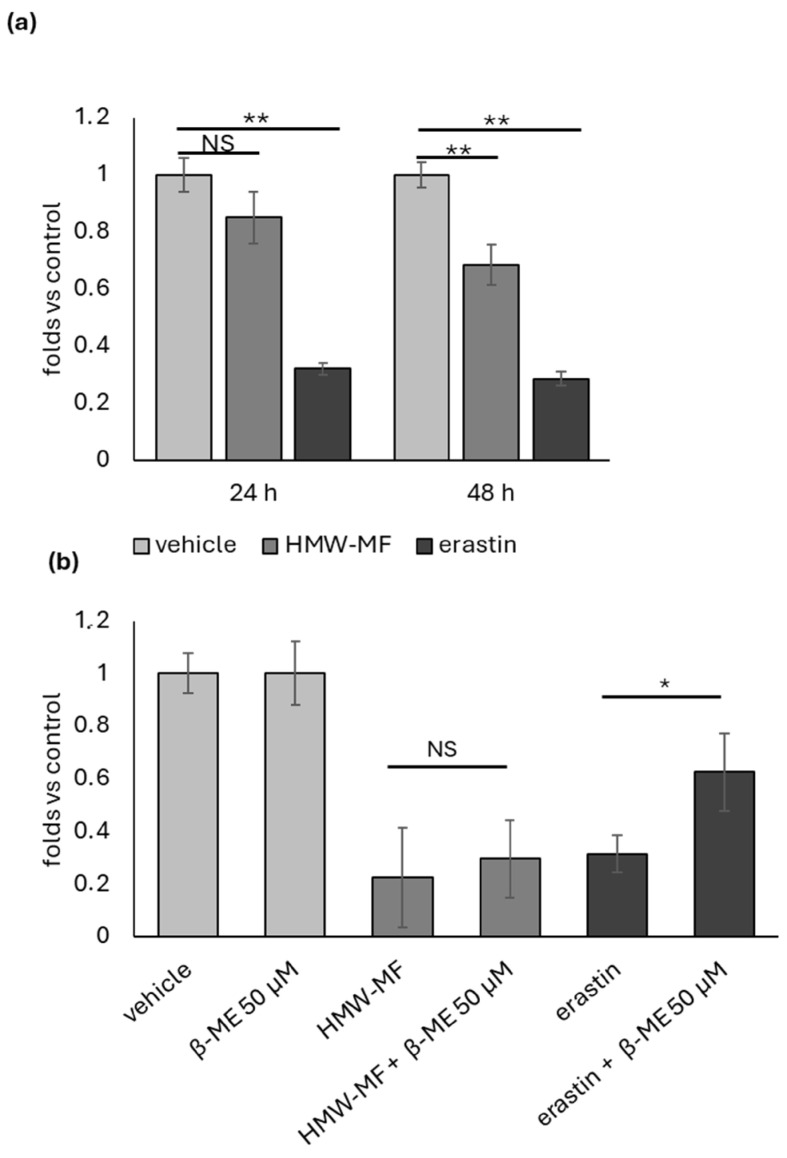
Analysis of GSH intracellular content and of the effect of β-ME co-treatment on HMW-MF- and erastin-induced reduction in cell growth. (**a**) Relative quantification of intracellular GSH through OPA fluorometric assay. Each bar represents the mean ± SD of five independent samples normalized versus the corresponding controls to which an arbitrary value of 1 was attributed. ** = *p* < 0.01 calculated using two-way ANOVA; NS = not significant. (**b**) Representative crystal violet assay performed 48 h after treatment with HMW-MF or erastin in the presence or absence of 50 µM β-ME. Each bar represents the mean ± SD of five independent samples normalized versus the corresponding controls to which an arbitrary value of 1 was attributed. * = *p* < 0.05, calculated using one-way ANOVA; NS, not significant.

Previous data report a significative redox unbalance in mucus-treated cells [12]. Consistently, the o-phthalaldehyde (OPA) fluorometric assay indicated a progressive decrease in GSH levels, becoming statistically significant at 48 h. However, the decrease in GSH levels induced by HMW-MF treatment was markedly lower than that observed following erastin exposure (Figure 2a).

Erastin belongs to class I ferroptosis-inducing novel compounds (FINs) and triggers ferroptosis by directly inhibiting the Xc^−^ glutamate/cysteine antiporter. Blockade of this transporter leads to a gradual depletion of cysteine, compromising GSH synthesis and weakening the cell’s antioxidant defenses. Erastin-induced ferroptosis can be attenuated by co-treatment with β-mercaptoethanol (β-ME), which reacts with cystine to form a mixed disulfide that is transported into cells via System L, rapidly replenishing cysteine [13].

**Figure 3:**
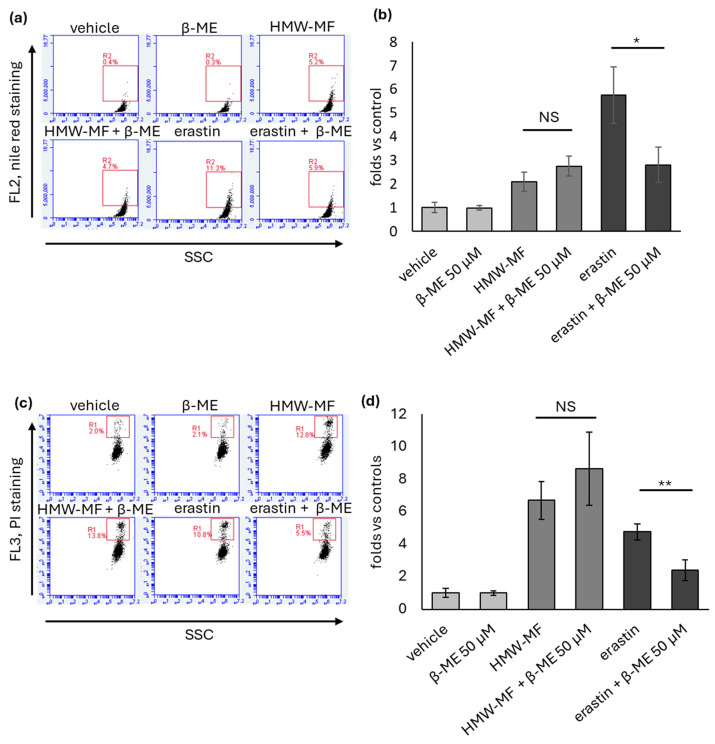
Effect of β-mercaptoethanol co-treatment on HMW-MF- and erastin-induced cytotoxic activity. (**a**) Representative flow cytometry plots from the Nile red incorporation assays. Events included in the R2 box were considered positive. SSC, side scatter channel. (**b**) Quantification of cells included in the R2 box detected in a representative experiment 48 h after treatment with mucus or erastin, in the presence or absence of 50 µM β-ME. Each bar represents the mean ± SD of five independent samples normalized versus the corresponding controls to which an arbitrary value of 1 was attributed. * = *p* < 0.05 calculated using one-way ANOVA; NS, not significant. (**c**) Representative flow cytometry plots from the PI exclusion assays. Events included in the R1 box were considered positive. SSC, side scatter channel. (**d**) Quantification of cells included in the R1 box detected in a representative experiment 48 h after treatment with HMW-MF or erastin in the presence or absence of 50 µM β-ME. Each bar represents the mean ± SD of five independent samples normalized versus the corresponding controls to which an arbitrary value of 1 was attributed. ** = *p* < 0.01, calculated using one-way ANOVA, NS, not significant.

Based on the finding that GSH is significantly reduced upon HMW-MF treatment, we wondered whether HMW-MF acts as an Xc^−^ inhibitor. To this aim, NCI-H358 cells were co-treated with HMW-MF and 50 µM β-ME. As positive controls, cells were treated with erastin alone or in combination with β-ME. As shown in Figure 2 and Figure 3, co-treatment with β-ME partially rescued the cytotoxic effect of erastin but had no protective effect against HMW-MF-induced cytotoxicity. Indeed, no differences were recorded in the number of cells detected by crystal violet assay (Figure 2b), in the accumulation of lipid droplets detected by Nile red incorporation assay (Figure 3a,b), or in the percentage of death cells detected by PI exclusion assay (Figure 3c,d) between HMW-MF and HMW-MF + β-mercaptoethanol-treated cells.

### 2.3. HMW-MF Induces an Early PERK–ATF4–CHAC1 Response

**Figure 4:**
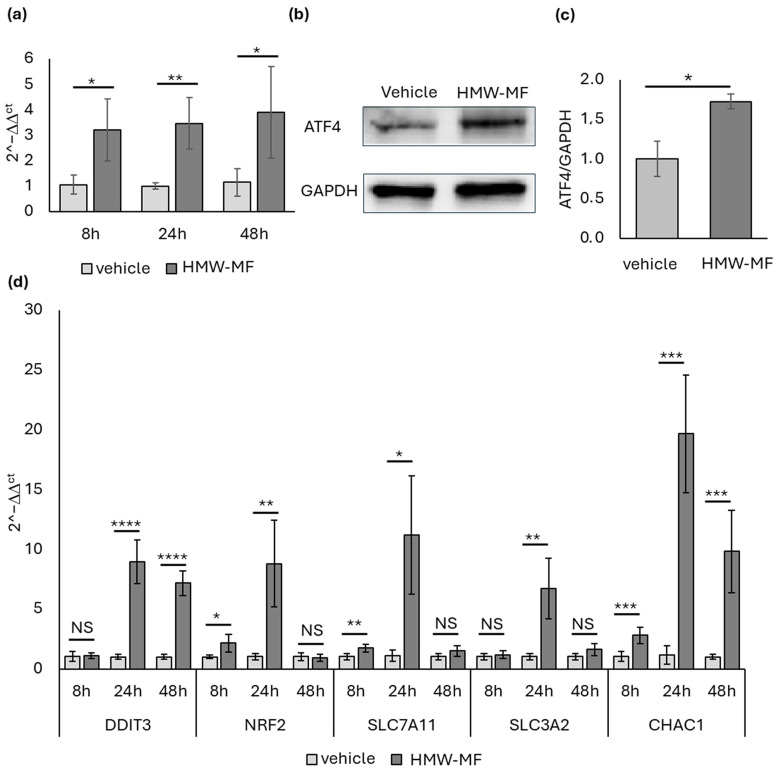
Analysis of the PERK branch of UPR. (**a**) Relative quantification of *ATF4* mRNA level from a representative real-time RT-PCR experiment. Each bar represents the mean ± SD of five independent samples normalized versus the corresponding controls to which an arbitrary value of 1 was attributed. * = *p* < 0.05, ** = *p* < 0.01, calculated using two-way ANOVA. (**b**) Representative Western blots showing the expression of ATF4 and GAPDH in HMW-MF- and vehicle-treated samples. (**c**) Densitometric quantification of ATF4 protein levels normalized to GAPDH. Each bar represents the mean ± SD of five independent samples normalized versus the corresponding controls to which an arbitrary value of 1 was attributed. * = *p* < 0.05, calculated using Student’s *t*-test. (**d**) Relative quantification of *DDIT3*, *NRF2*, *SLC7A11*, *SLC3A2*, and *CHAC1* mRNA levels from a representative real-time RT-PCR experiment. Each bar represents the mean ± SD of five independent samples normalized versus the corresponding controls to which an arbitrary value of 1 was attributed. * = *p* < 0.05, ** = *p* < 0.01, *** = *p* < 0.005, **** = *p* < 0.001 calculated using two-way ANOVA; NS, not significant.

Real-time RT-PCR analyses revealed a significant induction of *ATF4* mRNA as early as 8 h after the HMW-MF treatment, which was maintained at 24 h and 48 h (Figure 4a), indicating early activation of the PERK branch of the UPR. Consistently, ATF4 protein levels were significantly increased at 48 h (Figure 4b,c).

In line with ATF4 activation, its downstream target *DDIT3* was significantly upregulated at 24 h and 48 h (Figure 4d). Moreover, *NRF2* mRNA levels increased starting from 8 h and remained elevated at 24 h (Figure 4d), accompanied by the upregulation of its target genes *SLC7A11* and *SLC3A2* (Figure 4d). Notably, the expression of *NRF2* and its targets returned to basal levels at 48 h.

Importantly, *CHAC1* mRNA expression was significantly increased at 8, 24, and 48 h (Figure 4d), supporting an early activation of the PERK–ATF4–CHAC1 axis.

### 2.4. Late Activation of the IRE1α–XBP1 Pathway Without ATF6 Involvement

**Figure 5:**
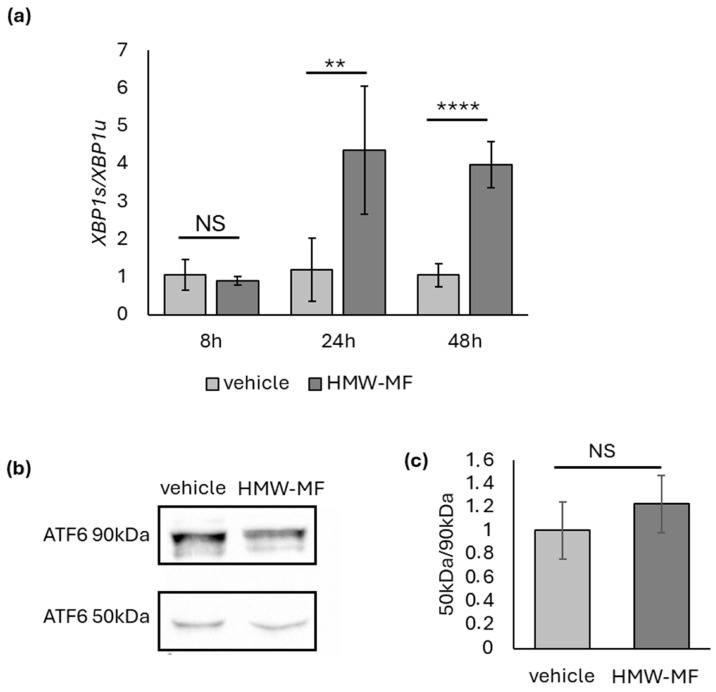
Analysis of the activation of the IRE1α and ATF6 arms of the UPR pathway. (**a**) Quantification of the *XBP1s/XBP1u* mRNA ratio. Each bar represents the mean ± SD of five independent samples normalized versus the corresponding controls to which an arbitrary value of 1 was attributed. ** = *p* < 0.05, **** = *p* < 0.001, calculated using two-way ANOVA; NS, not significant. (**b**) Representative Western blot showing ATF6 expression in HMW-MF- and vehicle-treated samples. (**c**) Quantification of the cleaved (50 kDa)/uncleaved (90 kDa) ATF6 ratio from five independent experiments. Each bar represents the mean ± SD of five independent samples normalized versus the corresponding controls to which an arbitrary value of 1 was attributed. NS, not significant; calculated using Student’s *t*-test.

To further characterize UPR activation, we evaluated the IRE1α and ATF6 branches. Real-time RT-PCR analysis showed a significant increase in the spliced/unspliced XBP1 ratio (*XBP1s/XBP1u*) at 24 h and 48 h after treatment, whereas no changes were detected at 8 h (Figure 5a), indicating a delayed activation of the IRE1α pathway.

**Figure 6:**
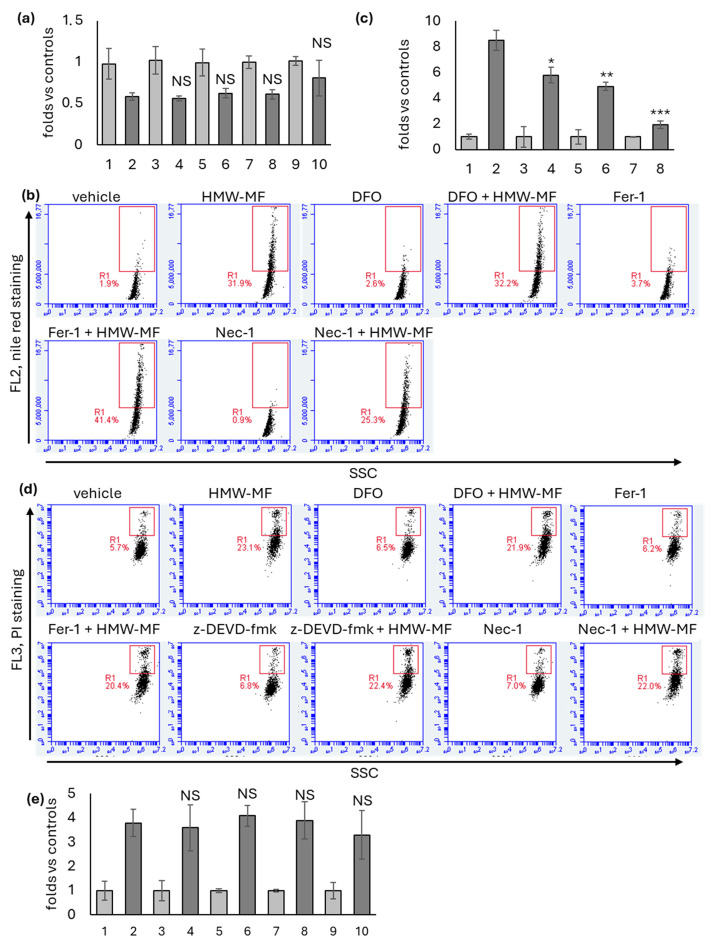
Effect of ferroptosis, caspase 3, and necroptosis inhibitors on HMW-MF cytotoxic activity. (**a**) Representative crystal violet assay performed 48 h after treatment with HMW-MF alone or in combination with the inhibitors. Each bar represents the mean ± SD of five independent samples. 1: vehicle; 2: HMW-MF; 3: DFO 12.5 µM; 4: DFO 12.5 µM + HMW-MF; 5: Fer-1 5 µM; 6: Fer-1 5 µM + HMW-MF; 7: z-DEVD-fmk 1 µM; 8: z-DEVD-fmk 1 µM + HMW-MF; 9: Nec-1 40 µM; 10: Nec-1 40 µM + HMW-MF. One-way ANOVA was applied to evaluate statistically significant differences between HMW-MF samples and HMW-MF + inhibitors samples. NS, not significant. (**b**) Representative flow cytometry plots from the Nile red incorporation assays. Events included in the R1 box were considered positive. SSC, side scatter channel (**c**) Quantification of cells included in the R1 box detected in a representative experiment 48 h after treatment with HMW-MF alone or in combination with the inhibitors. Each bar represents the mean ± SD of five independent samples normalized versus the corresponding controls to which an arbitrary value of 1 was attributed. 1: vehicle; 2: HMW-MF; 3: DFO 12.5 µM; 4: DFO 12.5 µM + HMW-MF; 5: Fer-1 5 µM; 6: Fer-1 5 µM + HMW-MF; 7: Nec-1 40 µM; 8: Nec-1 40 µM + HMW-MF; one-way ANOVA was applied to evaluate statistically significant differences between HMW-MF samples and HMW-MF + inhibitor samples. * = *p* < 0.05; ** = *p* < 0.01; *** = *p* < 0.005. (**d**) Representative flow cytometry plots from the PI exclusion assays. Events included in the R1 box were considered positive. SSC, side scatter channel. (**e**) Quantification of cells included in the R1 box detected 48 h after treatment with HMW-MF alone or in combination with the inhibitors. Each bar represents the mean ± SD of five independent experiments. The data of each experiment were normalized versus the corresponding controls to which an arbitrary value of 1 was attributed. 1: vehicle; 2: HMW-MF; 3: DFO 12.5 µM; 4: DFO 12.5 µM + HMW-MF; 5: Fer-1 5 µM; 6: Fer-1 5 µM + HMW-MF; 7: z-DEVD-fmk 1 µM; 8: z-DEVD-fmk 1 µM + HMW-MF; 9: Nec-1 40 µM; 10: Nec-1 40 µM + HMW-MF. One-way ANOVA was applied to evaluate statistically significant differences between HMW-MF samples and HMW-MF + inhibitor samples. NS, not significant.

By contrast, no increase in the cleaved active form of ATF6 (50 kDa) was observed 24 h after treatment (Figure 6b,c), suggesting that this branch is not significantly engaged under these conditions.

### 2.5. Pharmacological Inhibition of Canonical Cell Death Pathways Does Not Rescue Mucus-Induced Cytotoxicity

Previous ultrastructural analyses [12] revealed that mucus-treated cells display morphological features consistent with multiple cell death pathways. In particular, we observed lipid droplet accumulation and shrunken mitochondria, both indicative of a ferroptotic-like process [13]; small discontinuities of the plasma membrane, described in necroptosis [14]; and, in some cells, evident chromatin condensation and margination, typical of apoptosis [15].

To gain insight into the predominant mode of cell death, we performed co-treatment experiments using pharmacological inhibitors targeting distinct regulated cell death pathways. Deferoxamine (DFO), an iron-chelating agent, and ferrostatin-1 (Fer-1), a small synthetic molecule that prevents the propagation of lipid peroxidation within membranes, were employed to interfere with ferroptosis [13]; the caspase 3 inhibitor z-DEVD-fmk was used to inhibit caspase 3-dependent cell death modality [16] and necrostatin-1 (Nec-1) was applied to block necroptosis [17].

Following co-treatment with HMW-MF and each inhibitor, we quantified the number of cells through the crystal violet assay (Figure 6a), we assessed lipid accumulation through Nile red staining (Figure 6b,c), and we quantified the number of dead cell through PI incorporation (Figure 6d,e).

Despite DFO, Fer-1 and Nec-1 produced a significant and reproducible reduction in lipid accumulation (Figure 6b,c), no protective effect was observed for all the inhibitors regarding the total number of cell evaluated by crystal violet assay (Figure 6a), and the effects were not consistently reproducible across independent experiments for PI exclusion assays. Indeed, only occasionally in some experiments, we observed a modest reduction in the percentage of PI-positive cells in co-treated samples compared with HMW-MF alone and overall, none of the inhibitors was able to significantly or reliably rescue cell viability (Figure 6d,e).

## 3. Discussion

Exploiting oxidative stress represents an established approach in cancer therapy, as malignant cells operate close to the threshold of redox tolerance [18]. Such perturbations are tightly linked to endoplasmic reticulum stress and to activation of UPR, a central adaptive pathway that can shift from pro-survival to pro-death signaling under conditions of sustained stress [19].

In our experimental model, the cytotoxic activity exerted by planarian mucus is clearly ROS-dependent, as demonstrated by its rescue upon treatment with the antioxidant N-acetylcysteine [12]. This observation identifies oxidative stress as a central mediator of mucus-induced cell death and prompted us to directly investigate intracellular redox homeostasis.

To this end, we measured GSH, a key determinant of cellular redox buffering capacity. The HMW-MF treatment induced a significant decrease in intracellular GSH levels, consistent with the previously reported accumulation of reactive oxygen species [12]. Importantly, this depletion does not appear to result from the inhibition of the System Xc^−^ transporter. Indeed, unlike erastin, the cytotoxic effect of HMW-MF was not rescued by β-mercaptoethanol, indicating that cystine import through Xc^−^ is not the primary target. Consistently, although NRF2 and its target genes *SLC7A11* and *SLC3A2* were upregulated following HMW-MF exposure, this likely reflects a compensatory antioxidant response rather than a direct inhibition of the transporter, as observed with erastin [20,21].

Notably, *CHAC1* transcription was markedly upregulated following HMW-MF treatment, preceding the observed decrease in GSH levels. *CHAC1* encodes γ-glutamyl cyclotransferase that degrades GSH and is recognized as a pro-oxidant effector under stress conditions [22]. Therefore, increased CHAC1 expression may contribute to GSH depletion. However, this interpretation remains speculative and requires direct experimental validation, such as assessment of CHAC1 enzymatic activity or loss-of-function approaches. Accordingly, CHAC1 should be regarded as a candidate mediator rather than a demonstrated driver of the observed phenotype. Importantly, CHAC1 expression is known to be regulated by ATF4.

Consistent with this, HMW-MF-treated cells exhibited an early and sustained increase in ATF4 levels, occurring when *CHAC1* transcript levels begin to rise moderately and well before the onset of overt cytotoxic effects. This is indicative of the early activation of the PERK branch UPR [23]. In addition to PERK–ATF4 signaling, HMW-MF treatment also engages the IRE1α branch of the UPR, as demonstrated by the increased splicing of *XBP1* mRNA at later time points. This temporal pattern suggests a progressive UPR activation, with an early PERK-driven response followed by a delayed involvement of the IRE1α pathway. By contrast, the ATF6 branch was not activated, indicating a selective engagement of UPR signaling.

Within this framework, NRF2 upregulation may likely represent an adaptive response aimed at restoring redox homeostasis. ATF4 has been reported to transcriptionally activate NRF2, which in turn induces the expression of SLC7A11 and SLC3A2. However, this compensatory response appears to be transient and largely confined to the first 24 h after treatment, during which increased antioxidant capacity may partially counterbalance GSH depletion and delay the onset of cytotoxic effects. Later (48 h), NRF2 and its target genes return toward baseline levels, in parallel with a further reduction in GSH and the emergence of overt cellular alterations.

Based on these observations, we propose a working model in which: (i) mucus induces an early activation of the PERK branch of the UPR; (ii) this leads to increased ATF4-dependent CHAC1 expression; (iii) CHAC1-mediated GSH degradation contributes to intracellular GSH depletion; (iv) the resulting redox imbalance promotes oxidative stress and the activation of a robust UPR involving both PERK and IRE1α branches; and (v) sustained ATF4 activity further enhances CHAC1 expression, establishing a positive feedback loop that amplifies oxidative damage and ultimately drives cytotoxicity.

Nevertheless, the precise mode of cell death remains incompletely defined. None of the inhibitors tested fully restored cell viability, suggesting that HMW-MF-induced cytotoxicity may not be attributable to a single canonical death pathway. However, these results should be interpreted with caution as DFO was applied only as a pre-treatment and not maintained throughout the assay, and the caspase inhibitor used (z-DEVD-fmk) does not ensure broad inhibition of all caspase activities. Although we confirmed that DFO is functionally active under our experimental conditions (see Figure S1), incomplete or transient pathway inhibition may have limited the extent of rescue.

Within these constraints, two non-mutually exclusive scenarios can be envisaged: HMW-MF may activate multiple, partially redundant cell death programs, or it may trigger a non-canonical form of regulated cell death. Such functional plasticity would be consistent with the simultaneous activation of multiple stress response pathways.

Although technical limitations still prevent biochemical characterization of the HMW-MF active component(s), and thus preclude definitive attribution of the observed effects to a specific molecular entity, the available evidence highlights planarian mucus as a promising natural product with pleiotropic cytostatic and cytotoxic properties. The ROS-dependent positive feedback loop involving selective UPR activation, CHAC1 induction, and GSH depletion likely represents a key component of its cytotoxic mechanism. Further studies will be required to validate this model and to identify the molecular nature of the active component(s).

## 4. Materials and Methods

### 4.1. Planarian Rearing

Planarians of the species *Dugesia japonica* (clonal strain GI) were raised in planarian water according to what described in Gambino and co-workers [12].

### 4.2. Planarian Mucus Preparation, Quantification, Storage, Fractionation, and Treatment

Planarian mucus was prepared according to the procedure described by Gambino and co-workers [12]. Briefly, total mucus was prepared from approximately 200 specimens following stimulation with 0.025% Triton X-100. The secreted mucus was manually collected, mixed 1:1 with 5% N-acetylcysteine (NAC), and concentrated using 3 kDa MWCO protein concentrators to restore the initial volume. The sample was then diluted 10-fold with PBS (final NAC concentration 0.25%) and concentrated again to the original volume. Each new extraction was quantified using the phenol–sulfuric acid method for carbohydrate estimation, as described by Masuko and co-workers [24].

Each sample was then tested to determine its EC_50_ value using the crystal violet assay after 48 h of treatment. The mucus was aliquoted and stored at −20 °C. Most treatments were performed at a dose corresponding to four times the EC_50_. As the mucus preparation protocol inherently results in trace amounts of NAC in the final preparation [12], all control conditions included an equivalent NAC concentration (vehicle) at the final concentration of 0.12 mM. Importantly, this concentration is well below levels commonly associated with antioxidant effects. Some mucus aliquots were denatured in Laemmli buffer, boiled for 5 min, separated on 10% SDS–polyacrylamide gel, and stained with Coomassie Brilliant Blue R-250 (Bio-Rad Laboratories, Bio-Rad, Hercules, CA, USA).

### 4.3. Cell Cultures and Treatments

The human bronchioalveolar carcinoma cell line NCI-H358 was kindly provided by Doctor Laura Poliseno from the Italian National Research Council of Pisa. The cells were cultured in RPMI 1640 medium w/o L-glutamine (yourSial, S.I.A.L. Srl, Rome, Italy) supplemented with 10% heat-inactivated fetal bovine serum (yourSial, S.I.A.L. Srl, Rome, Italy), 2 mM L-glutamine, 100 units/mL penicillin, and 100 μg/mL streptomycin (yourSial, S.I.A.L. Srl, Rome, Italy). The cells were cultured at 37 °C in a humidified atmosphere containing 5% CO_2_ and 95% air and subjected to a 1:3 split every 3 days. DFO was purchased from Cayman Chemical Company (Ann Arbor, MI, USA) and diluted in DMSO to obtain stock solution at the final concentration of 3.8 mM. Stock solution was aliquoted and stored at minus 20. The cells were pre-treated with 12.5 µM DFO for 2 h; then, due to its high toxicity, we washed out the DFO and treated cells with mucus or vehicle for 48 h. This treatment modality was able to reduce cytotoxicity induced by erastin (the canonical ferroptosis inducer) (Figure S1). Erastin was purchased from Merk KGaA (Darmstadt, Germany) and diluted in DMSO to obtain a stock solution at the final concentration of 20 mM. The stock solution was aliquoted and stored at minus 20. The cells were exposed to erastin (5 µM) for 48 h. Fer-1 was purchased from Cayman Chemical Company (Ann Arbor, MI, USA) and diluted in DMSO to obtain stock solution at the final concentration of 9.5 mM. The stock solution was aliquoted and stored at minus 20. The cells were pre-treated with 5 µM Fer-1 for 1 h, followed by the addition of mucus or vehicle for 48 h. Nec-1 was purchased from Cayman Chemical Company (Ann Arbor, MI, USA) and diluted in DMSO to obtain stock solution at the final concentration of 19.3 mM. The stock solution was aliquoted and stored at minus 20. The cells were pre-treated with 40 µM necrostatin-1 for 30 min, followed by the addition of mucus or vehicle for 48 h. z-DEVD-fmk was purchased from Cayman Chemical Company (MI, USA) and diluted in DMSO to obtain stock solution at the final concentration of 0.75 mM. Stock solution was aliquoted and stored at −20 °C until use. The cells were pre-treated with 1 µM z-DEVD-fmk for 2 h, followed by the addition of mucus or vehicle for 48 h.

### 4.4. Protein Extraction and Western Blotting

The NCI-H358 cells were resuspended in 100 µL of lysis buffer (K_2_HPO_4_ 100 mM, KCl 1.15%, EDTA 1 mM pH 7.4). The mixture was incubated for 30 min on ice, then subjected to three freeze–thaw cycles, and finally centrifuged at 12,000 rpm for 10 min at 4 °C. The supernatant was then quantified using Pierce™ BCA Protein Assay Kit (Thermo Fisher Scientific, Waltham, MA, USA).

Protein extracts were either used for GSH quantification (see below) or for Western blot analysis.

For Western blotting, the samples were denatured in Laemmli buffer and boiled for 5 min, separated on 8–10% SDS–polyacrylamide gels, and electrotransferred to nitrocellulose membranes (Bio-Rad Laboratories, Bio-Rad, Hercules, CA, USA) using a Mini Trans-Blot Cell system (Bio-Rad Laboratories, Bio-Rad, Hercules, CA, USA).

The membranes were blocked at room temperature for 1 h using 5% fat-free milk (Merck KGaA, Darmstadt, Germany) in TBST, and then incubated overnight at 4 °C with anti-ATF4 (#11815, Cell Signaling, Danvers, MA, USA; rabbit monoclonal antibody, 1:1000 in 5% BSA in TBST) or anti-ATF6 antibodies (#sc-166660 Santa Cruz Biotechnology, Dallas, TX, USA; mouse monoclonal antibody, 1:750 in 3% milk in TBST). The detection of the primary antibody was performed using horseradish peroxidase-conjugated secondary antibodies and enhanced chemiluminescence reagents (Clarity Western ECL Substrate or Clarity Max Western ECL, Bio-Rad, Hercules, CA, USA). Densitometry analysis was performed using ImageLab 6.1 software (Bio-Rad Laboratories, Bio-Rad, Hercules, CA, USA). Normalization was performed using the internal loading control GAPDH (#21118, Cell Signaling, Danvers, MA, USA; rabbit polyclonal antibody, 1:3000 in 3% milk in TBST).

### 4.5. GSH Assay

Intracellular GSH levels were determined as previously described by [25]. Cell extracts obtained as detailed above were deproteinized by adding 10% trichloroacetic acid (TCA) and incubated for 30 min at 4 °C. An aliquot of the supernatant was combined 1:1 with o-phthalaldehyde (1 mg/mL in 10% methanol, *v*/*v*), mixed with GSH buffer (50 mM K_2_HPO_4_, 50 mM EDTA, pH 8.0), and incubated for 15 min at 37 °C. After centrifugation (625× *g* for 3 min), fluorescence was measured (λex = 350 nm, λem = 420 nm) using a fluorescence spectrometer (PerkinElmer LS-45, PerkinElmer Life and Analytical Sciences, Shelton, CT, USA).

### 4.6. Cell Viability Assays

Crystal violet assay, propidium iodide (PI) exclusion assay, and analysis of lipid content Nile red staining (Sigma-Aldrich, Merck, Darmstadt, Germany) were performed according to the procedures described by Gambino et al. [12].

### 4.7. RNA Extraction, Quantification, and Retrotranscription

RNA was extracted using TRIzol reagent (Thermo Fisher Scientific, Waltham, MA, USA), following the manufacturer’s instructions. RNA was subsequently quantified using Nanodrop Lite (Thermo Fisher Scientific, Waltham, MA, USA). A total of 0.25 µg of RNA was retrotranscribed using RevertAid First Strand cDNA Synthesis Kit (Thermo Fisher Scientific, Waltham, MA, USA), following the manufacturer’s instructions and using a GeneAmp^®^ PCR System 2700 Thermal Cycler (Applied Biosystems—Thermo Fisher Scientific, Waltham, MA, USA).

### 4.8. Real-Time PCR

Real-time PCR was performed with GoTaq^®^ qPCR Master Mix amplification mixture (Promega Corporation, Madison, WI, USA) on a QuantStudio™ 5 Real-Time PCR System (Thermo Fisher Scientific, Waltham, MA, USA) using the oligonucleotides according to Table S1. A melting curve was performed after each PCR reaction to confirm the specificity of the primers. All reactions were performed in duplicate. The data were analyzed using Design & Analysis Software 2.8.0 (Thermo Fisher Scientific, Waltham, MA, USA), using *HPRT* and *GAPDH* as reference genes.

### 4.9. Statistical Analysis

Normality of data distribution was evaluated using the Shapiro–Wilk test. As all datasets showed a normal distribution, statistical differences between experimental groups were analyzed using Student’s *t*-test, one-way ANOVA or two-way ANOVA followed by selected pairwise comparisons corrected for multiple testing using the Šidák method where appropriate. A *p*-value < 0.05 was considered indicative of statistical significance. Each experiment was analyzed independently, and the reproducibility of the results was confirmed in three separate experimental replicates. When treated groups did not share a common control, the data for each group were normalized to the mean value of their respective control prior to comparison. Data visualization was performed using Microsoft Excel. Statistical analyses were calculated with the GraphPad Prism software 10 (GraphPad Software Inc., San Diego, CA, USA). EC_50_ values were determined with GraphPad Prism software by applying a nonlinear regression analysis based on the inhibitor vs. response (three-parameter) model.

## Data Availability

All data generated or analyzed during this study are included in this manuscript.

## Supplementary Materials

The following supporting information can be downloaded at: https://www.mdpi.com/article/10.3390/ijms27104324/s1.
