# Supplementary material for: A High-Molecular-Weight Fraction of Planarian Mucus Triggers UPR-Linked Cell Death Pathway in Human Bronchioalveolar Carcinoma Cell Line NCI-H358
Source: Int J Mol Sci. 2026 May 12;27(10):4324. doi: 10.3390/ijms27104324 (PMC13206961; doi:10.3390/ijms27104324)
Supplement: Supplementary file 1 [file ijms-27-04324-s001.zip › ijms-4208925-supplementary.pdf]

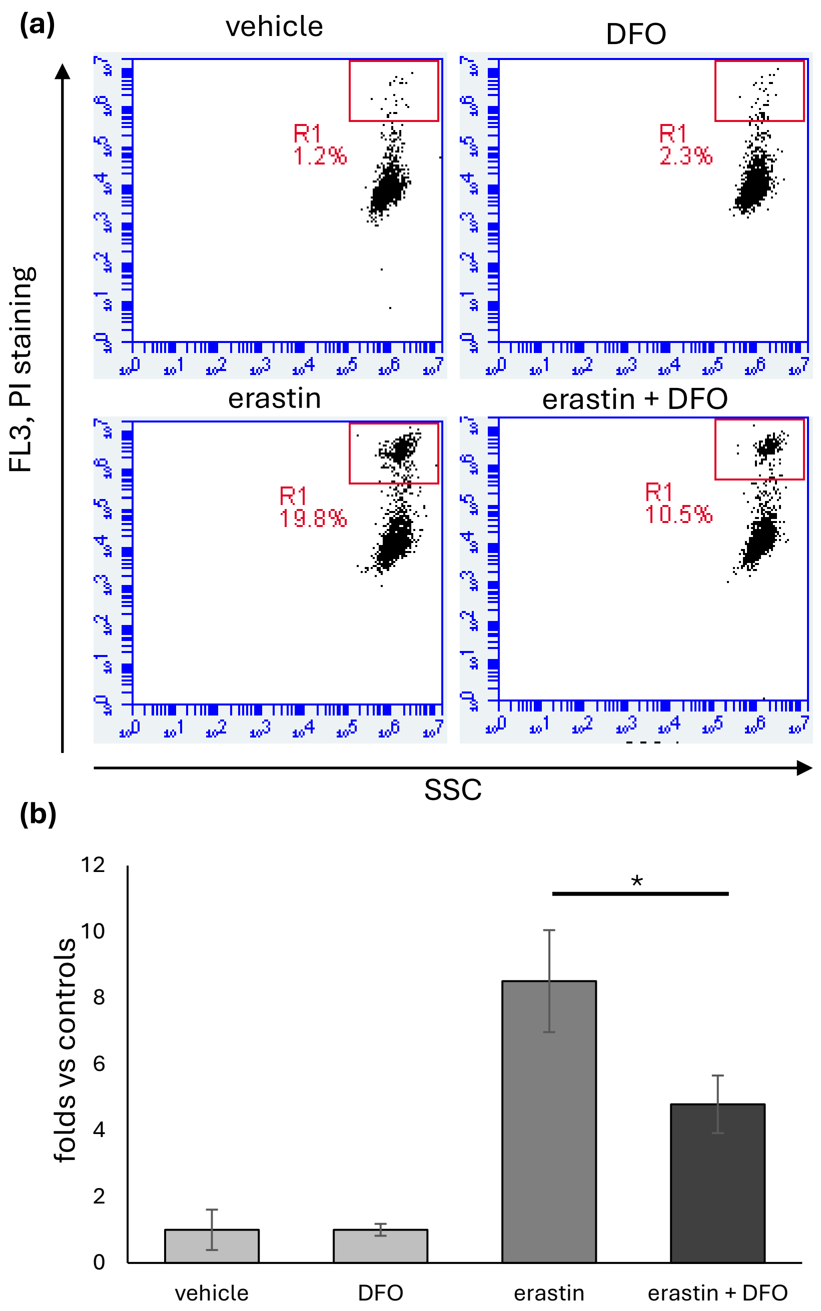

Figure S1: **Effect of DFO pre-treatment on erastin cytotoxic activity** (**a**) Representative flow cytometry plots from the PI exclusion assays. Events included in the R1 box were considered positive. SSC, Side scatter channel. (**b**) Quantification of cells included in the R1 box detected in a representative experiment 48 h after treatment with erastin, in the presence or absence of 12,5 μM DFO pre-treatment. Each bar represents the mean ± SD of five independent samples normalized versus the corresponding controls to which an arbitrary value of 1 was attributed. * = $p<0.05$. One-way ANOVA was applied to evaluate statistically significant differences between erastin samples and erastin + inhibitors samples.

Acronym	Full Name	Oligonucleotides (5'→3')
ATF4	Activating Transcription Factor 4	for: GTCCCTCCAACAACAGCAAG<br>rev: ACTTTCTGGGAGATGGCCAA
DDIT3	DNA Damage Inducible Transcript 3 (CHOP)	for: TGTTAAAGATGAGCGGGTGG<br>rev: AAAGGCAATGACTCAGCTGC
NRF2	Nuclear factor erythroid 2–related factor 2	for: GAGAGCCCAGTCTTCATTGC<br>rev: TTGGCTTCTGGACTTGGAAC
SLC7A11	Solute Carrier Family 7 Member 11	for: TCCGATCTTTGTTGCCCTCT<br>rev: GACTGTCGAGGTCTCCAGAG
SLC3A2	Solute Carrier Family 3 Member 2	for: CCTTGTGCTGGGTCCAATTC<br>rev: AGTTCTCACCCCGGTAGTTG
HPRT	Hypoxanthine-Guanine Phosphoribosyltransferase	for: CCTGGCGTCGTGATTAGTG<br>rev: GCCTCCCATCTCCTTCATC
XBP1s	X-box Binding Protein 1, spliced form	for: CTGAGTCCGAATCAGGTGCAG<br>rev: GGCTGGTAAGGAACTGGGTC
XBP1u	X-box Binding Protein 1, unspliced form	for: CACTCAGACTACGTGCACCT<br>rev: CACTCAGACTACGTGCACCT
CHAC1	ChaC glutathione-specific γ-glutamylcyclotransferase 1	for: CGCTGTGGATTTTCGGGTAC<br>rev: ATCTTCAAGGAGCGTCACCA
GAPDH	Glyceraldehyde-3-Phosphate Dehydrogenase	for: AAGGTGAAGGTCGGAGTCAAC<br>rev: GTTCTCAGCCTTGACGGTGC

Table S1: Sequence of primers.
